# Evaluating the role of human papillomaviruses in conjunctival neoplasia

**DOI:** 10.1038/sj.bjc.6602921

**Published:** 2006-01-10

**Authors:** M L Tornesello, M L Duraturo, K M Waddell, B Biryahwaho, R Downing, S Balinandi, S B Lucas, L Buonaguro, F M Buonaguro

**Affiliations:** 1 Viral Oncology and AIDS Reference Centre, National Cancer Institute ‘Fond. Pascale’, Cappella Cangiani, I-80131 Naples, Italy; 2 Uganda Eye Project, PO Box 4008, Kampala, Uganda; 3 Uganda Virus Research Institute, PO Box 49, Entebbe, Uganda; 4 Department of Histopathology, Guy's, King's & St Thomas' School of Medicine, St Thomas' Hospital, London SE1, UK

**Keywords:** conjunctiva squamous cell carcinoma, conjunctival intraepithelial neoplasia, papillomavirus, human immunodeficiency virus, Uganda

## Abstract

Mucosal, cutaneous and *Epidermodysplasia verruciformis (EV)*-related human papillomaviruses (HPVs) were searched by broad-spectrum PCR in 86 conjunctival neoplasia biopsies and 63 conjunctival non-neoplastic control tissue from Ugandan subjects. Seven different EV-related HPV types, including a putative new HPV, and two mucosal HPVs were detected in 25% (14 out of 56) of HIV-positive, in 10% (three out of 30) of HIV-negative conjunctival neoplasia samples, and rarely (0–1.6%) in control subjects. The absence of high-risk HPVs and the low detection frequency of EV-related HPV types in more advanced tumour stages (10%) raise doubts about their role in conjunctival carcinomas.

Conjunctival squamous cell neoplasia embraces ocular surface epithelial premalignant dysplasias and invasive carcinoma (Blodi, 1973). The histological staging is classified, as in cervical squamous cell neoplasia, by the thickness of epithelial dysplastic changes and the tumour invasion into the *substantia propria* (Waring *et al*, 1984). Although rare in Europe and USA, conjunctival neoplasia has been reported to be relatively common in regions of sub-Saharan Africa since the 1960s (Templeton, 1973). An emerging miniepidemic of conjunctival tumours has been described in Rwanda, Uganda and Tanzania in association with the epidemic of HIV infection since the 1980s (Ateenyi-Agaba, 1995; Waddell *et al*, 1996; Newton *et al*, 2002). The spread of HIV in Uganda, indeed, probably accounts for much of the approximately eight-fold increase in the incidence of conjunctival carcinoma observed during the last 20 years (Wabinga *et al*, 2000). So far the aetiology of conjunctival neoplasia is not adequately understood, although the association with HIV infection is most likely mediated by the immunosuppression, as in the other HIV-associated cancers (Beral and Newton, 1998).

High-risk mucosal human papillomavirus (HPV) types 16 and 18 have been detected in tissue biopsies of conjunctival neoplasias, although with highly variable results (Waddell *et al*, 1996; Buonaguro *et al*, 2000; Eng *et al*, 2002; Moubayed *et al*, 2004). More recently, Ateenyi-Agaba *et al* (2004) reported the presence of a discrete number of Epidermodysplasia verruciformis (EV) HPV types in 86% of invasive conjunctival squamous cell carcinoma, of which HIV serology was unknown, strongly suggesting their possible role also in such tumours.

We have analysed conjunctival neoplasia biopsies at different stages of malignancy (from conjunctival intraepithelial lesions grade 1 (CIN1) to invasive conjunctival squamous cell carcinoma (ICSCC)) to determine the prevalence of mucosal, cutaneous as well as EV-related HPV types in such lesions and their association with the tumour progression in HIV-positive as well as HIV-negative patients.

## PATIENTS AND METHODS

In total, 125 consecutive patients clinically diagnosed with conjunctival neoplasia at seven countrywide eye clinics in Southern Uganda, as part of the Ugandan Ruharo Eye Project, were enrolled as cases for this study between January 1997 and March 1999. The 125 cancer-free controls, frequency matched to the cases by sex and age (±10 years), were consecutively randomly enrolled from a pool of patients treated for benign eye lesions (i.e., pterygium) or eye injuries, within the same period of time, in the same eye clinics. All enrolled subjects, representing more than 95% of those approached, agreed to provide informed consent to participate. Blood serum samples were prospectively obtained from all cases (patients) as well as control subjects and analysed for HIV serology (ELISA screening followed by Western blot confirmation). The histological evaluation confirmed a total of 118 case biopsies as conjunctival neoplasias, the remaining seven were not confirmed as neoplastic tissue and excluded from the analysis.

DNA was extracted by digestion with proteinase K (150 *μ*g ml^−1^ at 60°C for 30 min) in lysis buffer (10 mM Tris-HCl pH 7.6, 5 mM EDTA, 150 mM NaCl, 1% SDS), followed by DNA purification, phenol and phenol–chloroform–isoamyl alcohol (25 : 24 : 1) extraction and ethanol precipitation in 0.3 M sodium acetate (pH 4.6). DNA quality test, performed by amplification with specific oligoprimers targeting a fragment of the exon 7 within the *TP53* gene, and DNA quantity analysis, evaluated by spectrophotometric measurements, rendered 86 cases and 63 controls suitable for the HPV detection. Testing for HPV was carried out with general-primer-mediated PCR using the following primer pairs: (a) MY09/MY11 (Resnick *et al*, 1990) and (b) GP5+/GP6+ (de Roda Husman *et al*, 1995), which preferentially amplify mucosal HPVs; (c) CP65/CP70 followed by CP66/CP69 for the amplification of EV-related HPV types (Berkhout *et al*, 1995); (d) FAP59/FAP64 for the amplification of 75 different types of mucosal, cutaneous and EV-related HPVs (Forslund *et al*, 1999). In addition, we used type-specific PCR targeting E6 genes of individual HPV16 and HPV38 types (Tornesello *et al*, 2000; Caldeira *et al*, 2003). Serial dilution (from one to 1000 copies) of 11 plasmid clones, containing HPV genomes representative of mucosal (HPV6, 11, 16, 18, 31, 33), cutaneous (HPV10), mucocutaneous (HPV2) and EV-related (HPV5, 8, 38) HPV types, were used as positive controls. Reaction mixtures without template DNA, included in every set of five clinical specimens, represented the negative controls. All amplified DNAs have been subjected to direct nucleotide sequence analysis following procedures previously described (Tornesello *et al*, 2000).

The data were analysed using *χ*^2^ test and, where appropriate, Fisher's exact test to calculate all *P*-values related to the differences between groups with Epi Info 6 Statistical Analysis System Software (6.04b, 1997, Centers for Disease Control and Prevention, USA). Differences were considered to be statistically significant when *P*-values were less than 0.05.

## RESULTS

This study enrolled 125 cases with clinical presentations compatible with conjunctival neoplastic lesions and 125 control subjects. Following histological typing and DNA quality/quantity assays, 86 confirmed conjunctival neoplasia and 63 cancer-free control samples were included in the HPV screening. Table 1 summarises the selected characteristics of the subjects. There were no significant differences between cases and controls in the distribution of age and sex, suggesting that the frequency matching on these two variables were adequate. The median age was 32 years (mean 34.5±11.5 years) for the cases, and 30 years (mean 35.6±10.7 years) for the controls. The HIV seroprevalence among cases was significantly higher (*P*=0.0095) than that observed in controls, confirming that HIV infection is a relevant risk factor in the pathogenesis of conjunctival carcinoma in this population (Table 1).

All 86 cases and 63 controls have been analysed by PCR using six primer sets to amplify mucosal, cutaneous as well as EV-related HPV types. Combining the results obtained with MY09/MY11, GP5+/GP6+, CP65/CP70-CP66/CP69 and HPV38 primer pairs, 18 samples were positive for the presence of HPVs (Table 1). No viral sequences were detected in either cases or control with FAP59/FAP64 and HPV16-specific primer pairs.

The HPV-positive samples comprise 17 (19.8%) conjunctival neoplasia and one (1.6%) control biopsy (*P*=0.0019). A total of 14 samples (25.0%), among the 56 conjunctival neoplasia from HIV-positive subjects, tested positive for HPV sequences (*P*=0.0312). Only 10% (three out of 30) of the neoplastic samples, from HIV-negative subjects, were HPV positive.

The histology of the 86 conjunctival neoplasia patients identified 29 (33.7%) lesions as ICSCC, 23 (26.7%) as CIN3, 18 (20.9%) as CIN2 and 16 (18.6%) as CIN1. The HPV distribution among cases, grouped by histological types, also stratified by HIV serology, are summarised in Table 2. The frequency of HPV infection among HIV-positive patients was 15% (three out of 20) in the ICSCC, 25% (three out of 12) in CIN3, 36.3% (four out of 11) in CIN2 and 33.3% (four out of 12) in CIN1. HPV sequences among HIV-negative patients were identified in 28.5% (two out of seven) of CIN2, 25.5% (one out of four) of CIN1, 4.1% (one out of 23) of controls and in none of the ICSCC and CIN3 samples. Therefore, HPV detection frequency decreased in more advanced disease stages.

Characterisation of the amplified HPV sequences allowed the identification of two mucosal HPVs (6 and 18 types) and six EV-related HPVs (14b, 20, 38, DL473, PPHL1FR and HPV100). A putative new HPV sequence (CJ198) was identified in one ICSCC and in one CIN1 sample.

## DISCUSSION

Overall, this study suggests that mucosal and EV HPVs do infect the conjunctival epithelium, but does not confirm a role for specific HPVs in the development of conjunctival carcinoma. Furthermore, the low frequency of EV HPV types in ICSCC compared to that found in preneoplastic lesions (CIN1-2) suggests that these HPV types are not involved in tumour progression.

These results are concordant with several others epidemiological studies, performed by broad consensus PCRs, clearly indicating that EV HPV types are commonly found in skin lesions (up to 90%) from immunosuppressed renal transplant recipients.

The high detection rates of HPV DNA both in healthy skin biopsies, plucked hairs or skin swab samples in immunocompetent (25–65% HPV-positivity) and in immunosuppressed (30–90% positivity) individuals may suggest an ubiquity nature of these viruses. Forslund *et al* (2004), analysing swab samples collected on the top of skin tumours, found HPV DNA in 69% of the samples against a 12% HPV positivity within the biopsies of the same tumours; they concluded that HPV positivity in skin tumour biopsies may merely reflect surface contamination by viral DNA or particles harboured in cells shed from infected healthy skin.

In conclusion, we report that in our study on conjunctival neoplasia from sub-Sahara region, cutaneous HPVs were not detected while mucosotropic HPV types have been rarely found. Moreover, EV-related HPVs were frequently identified in low-grade conjunctival neoplasia of HIV-positive patients but less commonly in invasive conjunctival carcinomas. Thus, HPV DNA found in conjunctival lesions might merely be a passenger.

## Figures and Tables

**Table 1:** Distribution of known variables between conjunctival neoplasia cases and controls

	**Conjunctival neoplasia *n* 86 (%)**	**Controls^a^ *n* 63 *(%)***	***P* value**
*Sex*			0.924
M	35 (40.7)	27 (42.8)	
F	51 (59.3)	36 (57.1)	
			
*Age*			0.955
≤30 years	39 (45.3)	28 (44.7)	
>30 years	47 (54.6)	35 (55.3)	
			
*HPV PCR*			0.002
Positive	17 (19.8)	1 (1.6)	
Negative	69 (80.2)	62 (98.4)	
			
*HIV serology* b			0.01
Positive	56 (65.1)	15 (38.5)	
Negative	30 (34.9)	24 (61.5)	

**HIV status**	**Conjunctival neoplasia *n* 86 (%)**	**Controls^a^ *n 39*^b^ *(%)***	***P* value**
*HIV positive*			0.031^c^
HPV-Pos	14 (25.0)	0 (0.0)	
HPV-Neg	42 (75.0)	15 (100)	
			
*HIV negative*			0.620^c^
HPV-Pos	3 (10.0)	1 (4.2)	
HPV-Neg	27 (90.0)	23 (95.8)	

a Two Pinguecula, one pterygium and one papilloma of the conjunctiva were included in the control group.

b A total of 24 control subjects with undetermined HIV serology were not included.

c Fisher exact test, two-tailed.

**Table 2:** HPV type distribution among the different grades of conjunctival neoplasia in HIV-positive and HIV-negative patients

**HPV genotype^a^**	**ICSCC HIV+ (*n*=20)**	**ICSCC HIV− (*n*=9)**	**CIN3 HIV+ (*n*=12)**	**CIN3 HIV− (*n*=11)**	**CIN2 HIV+ (*n*=11)**	**CIN2 HIV− (*n*=7)**	**CIN1 HIV+ (*n*=12)**	**CIN1 HIV− (*n*=4)**	**Controls HIV+ (*n*=15)**	**Controls HIV− (*n*=24)**	**Total (*n*=149)**
6	0	0	0	0	0	0	0	0	0	1^b^	1
14d	1	0	0	0	0	0	0	0	0	0	1
18	0	0	1	0	0	1	0	0	0	0	2
20	1	0	0	0	0	0	1	1	0	0	3
38	0	0	0	0	1	0	0	0	0	0	1
100^c^	0	0	2	0	1	0	1	0	0	0	4
DL473^c^	0	0	0	0	1	0	0	0	0	0	1
PPHL1FR^c^	0	0	0	0	1	0	0	0	0	0	1
CJ198^c^	1	0	0	0	0	0	1	0	0	0	2
Indeterm	0	0	0	0	0	1	1	0	0	0	2
											
Total	3 (15%)	0 (0%)	3 (25%)	0 (0%)	4 (36.3%)^d^	2 (28.5%)	4 (33.3%)^d^	1 (25.5%)	0 (0%)	1 (4.1%)	18 (12%)

a As determined by DNA sequencing.

b Identified in the single conjunctival papilloma sample included in this study.

c Putative new HPV type identified in this study (accession number DQ027814) and by others (DL473, AJ010825; PPHL1FR, L38388). HPV100 was previously identified as DL267, Z95966 (de Villiers, personal communication).

d Prevalence of HPVs is significantly higher in HIV-positive CIN1 and CIN2 cases in comparison to HIV-positive controls (*P*<0.05, Fisher's exact text).
